# Evidence for dynamic attentional bias toward positive emotion-laden words: A behavioral and electrophysiological study

**DOI:** 10.3389/fpsyg.2022.966774

**Published:** 2022-08-16

**Authors:** Jia Liu, Lin Fan, Jiaxing Jiang, Chi Li, Lingyun Tian, Xiaokun Zhang, Wangshu Feng

**Affiliations:** ^1^School of Foreign Studies, Hebei Normal University, Shijiazhuang, China; ^2^National Research Centre for Foreign Language Education, Beijing Foreign Studies University, Beijing, China; ^3^Artificial Intelligence and Human Languages Lab, Beijing Foreign Studies University, Beijing, China; ^4^Research Institute of Foreign Languages, Beijing Foreign Studies University, Beijing, China

**Keywords:** emotion-label words, emotion-laden words, valence, dot-probe task, ERP

## Abstract

There has been no consensus on the neural dissociation between emotion-label and emotion-laden words, which remains one of the major concerns in affective neurolinguistics. The current study adopted dot-probe tasks to investigate the valence effect on attentional bias toward Chinese emotion-label and emotion-laden words. Behavioral data showed that emotional word type and valence interacted in attentional bias scores with an attentional bias toward positive emotion-laden words rather than positive emotion-label words and that this bias was derived from the disengagement difficulty in positive emotion-laden words. In addition, an attentional bias toward negative emotion-label words relative to positive emotion-label words was observed. The event-related potential (ERP) data demonstrated an interaction between emotional word type, valence, and hemisphere. A significant hemisphere effect was observed during the processing of positive emotion-laden word pairs rather than positive emotion-label, negative emotion-label, and negative emotion-laden word pairs, with positive emotion-laden word pairs eliciting an enhanced P1 in the right hemisphere as compared to the left hemisphere. Our results found a dynamic attentional bias toward positive emotion-laden words; individuals allocated more attention to positive emotion-laden words in the early processing stage and had difficulty disengaging attention from them in the late processing stage.

## Introduction

The relationship between cognition and emotion has long been a subject of investigation in the fields of neuroscience, psychology, and linguistics (Dolan, 2002; Conrad et al., 2011; Fan et al., 2018). Recently, Hinojosa et al. (2020a) advanced the idea of affective neurolinguistics, which concentrates on the neural correlates between emotion and language. The dissociation between emotion-label and emotion-laden words is one of the primary issues in this field (Kissler, 2020; Liu et al., 2020; Wu et al., 2020b). Emotion-label words directly refer to emotions, such as *happy* and *sad*, while emotion-laden words do not label emotions directly, but elicit emotions in an indirect way, such as *success* and *punishment* (Pavlenko, 2008).

Behaviorally, there have been inconsistent conclusions regarding the dissociation between emotion-label and emotion-laden words. Emotion-label words were associated with shorter reaction times (RTs) than emotion-laden words in lexical decision tasks (LDTs; Kazanas and Altarriba, 2015, 2016a,b), affective Simon tasks (Altarriba and Basnight-Brown, 2010), masked priming emotional categorization tasks (ECTs; Bromberek-Dyzman et al., 2021) as well as tasks of free recall and ratings (El-Dakhs and Altarriba, 2019). For instance, Kazanas and Altarriba (2015) performed primed LDTs to investigate whether the processing of emotion-label stimuli was different from that of emotion-laden stimuli. Across both masked and unmasked LDTs, significant discrepancies in RTs and priming effects between emotion-label and emotion-laden words were observed, with slower RTs and attenuated priming effects for emotion-laden words relative to emotion-label words. The results suggested that these two types of emotional stimuli had critical processing differences. Findings from their follow-up study (Kazanas and Altarriba, 2016a) using a longer stimulus onset asynchrony (SOA; i.e., 1,000 ms) evidenced the differentiation between emotion-label and emotion-laden words, as they rediscovered the effects reported in their previous study with a shorter SOA (i.e., 250 ms). The above findings supported the theories of embodiment (Niedenthal, 2007; Barsalou, 2008; Horchak et al., 2014), which maintained that words with higher embodiment would be accessed faster than those with weaker embodiment. However, some other studies observed that there was no significant difference between the RTs to emotion-label and emotion-laden words (Vinson et al., 2014; Martin and Altarriba, 2017). For example, Martin and Altarriba (2017) used an LDT with hemifield presentation of words to investigate the cognitive mechanisms of emotion-label and emotion-laden words. The results indicated that there was no significant difference between the RTs to emotion-label and emotion-laden words.

Studies on the neural dissociation between these two types of emotional words could be summarized into four aspects. The first is the neural activation of emotion-label and emotion-laden words (Zhang et al., 2017, 2020; Wang et al., 2019). In an LDT, Zhang et al. (2017) observed that emotion-laden words were associated with reduced N170 than emotion-label words in the right hemisphere, which was correlated with emotion perception (Borod et al., 1998; Smith and Bulman-Fleming, 2005). Studies on word processing have documented that the component N170 indicated the attention allocated to the target (Zhao et al., 2012). An enhanced late positive component (LPC), which indicated the degree of elaborate processing (Citron, 2012; Zhang et al., 2014), was elicited in the right hemisphere as compared with the left hemisphere during the processing of negative emotion-label words. It was concluded that the cortical responses to these two types of emotional words were different during distinct time courses even when the abstractness of words was controlled (Wang et al., 2019).

The second aspect is the modulation of emotional word type on emotional conflict (Wu and Zhang, 2019; Zhang et al., 2019a,b). In a flanker task, Zhang et al. (2019b) found that negative emotion-laden words were responded to faster than negative emotion-label words. In the left hemisphere, negative emotion-label words elicited enhanced N2, which indicated attention allocation (Zhang et al., 2018), than negative emotion-laden words; in the right hemisphere, positive emotion-label words produced amplified N2 than positive emotion-laden words. Emotion-laden words evoked larger N2 under incongruent conditions (a target word surrounded by different words at upper and lower sites) than under congruent conditions (a target word surrounded by the same words at upper and lower sites). The findings indicated that emotional word type modulated the processing of emotional conflict.

The third concerns the different priming effects of emotion-label and emotion-laden words (Wu et al., 2020a,b, 2021). For example, Wu et al. (2020b) investigated how emotion-label and emotion-laden words modulate the processing of emotional pictures. When the valences of emotional words and pictures were different, the pictures primed by emotion-laden words elicited an enhanced early posterior negativity (EPN), which is a component sensitive to emotional information (Kissler et al., 2009), than those primed by emotion-label words. They concluded that these two types of emotional words produced different priming effects. The latest one explored the effects of processing levels on emotion-label and emotion-laden word processing (Liu et al., 2022). In ECTs and emotional Stroop tasks (ESTs), they found that valence and emotional word type interacted with each other only during the explicit processing of emotional words.

The processing differences between emotion-label and emotion-laden words might be associated with the attentional resources allocated to the words. A few behavioral studies have delved into this field. Experiment 1 in Knickerbocker and Altarriba (2013) adopted rapid serial visual presentation (RSVP) tasks to investigate the repetition blindness (RB) effect of neutral, negative emotion-label, and negative emotion-laden words. Compared to neutral and negative emotion-laden words, negative emotion-label words elicited a larger RB effect, demonstrating that in the early processing stage, emotion-label words captured more attentional resources than emotion-laden ones. Sutton and Altarriba (2011) used two dot-probe tasks to investigate the attentional bias toward neutral, positive emotion-label, and negative emotion-label words. In the first task, the word pairs were displayed for 180 ms. In the second task, the word pairs were displayed for 30 ms, followed by a 150-ms mask. Across tasks, there was no significant difference between the RTs to the probes following positive emotion-label words and neutral words, while the RTs to the probes following negative emotion-label words were shorter than those to neutral words. The findings showed an attentional bias toward negative emotion-label words.

Tasks such as LDTs and affective Simon tasks examined the semantic processing and affective conflict processing of words; in particular, the attentional bias toward emotional words could not be investigated. One of the optimal tasks to investigate attentional bias is the dot-probe task developed by MacLeod et al. (1986). In the task, two stimuli were simultaneously presented on opposite sides of the screen. One was neutral and the other was emotional (emotional pair). After the pair of stimuli, a probe was displayed in the location of the neutral (incongruent trial) or emotional (congruent trial) stimulus, and the participants were required to respond to the feature or location of the probe. If the participants automatically allocated more attention to one of the stimuli—probably the emotional one—the RTs for reacting to the dot following this emotional stimulus would be faster than the RTs for responding to the dot following the unattended neutral stimulus. Therefore, the RTs were a measure of attentional bias (*M*_incongruent_ − *M*_congruent_; *cf.* van Rooijen et al., 2017). Given this, an attentional bias index greater than 0 means a bias toward emotional words, while one less than 0 means a bias away from emotional words. To dissociate attentional orientation and attentional disengagement, Koster et al. (2004) added a neutral pair of stimuli—that is, both stimuli were neutral. Specifically, the dots appeared at either location of the neutral stimuli. These trials were neutral conditions. The mean RTs per condition were used to calculate attentional orientation (*M*_neutral_ − *M*_congruent_) and attentional disengagement (*M*_incongruent_ − *M*_neutral_) indices per condition (e.g., positive emotion-label, positive emotion-laden, negative emotion-label, and negative emotion-laden). An attentional orientation index greater than 0 indicates attentional vigilance toward emotional words, while one less than 0 indicates attentional avoidance of emotional words. An attentional disengagement index greater than 0 indicates difficulty in disengaging attention from emotional words, and one less than 0 means fast disengagement from emotional words.

The reliability of dot-probe tasks could be improved by combining them with the technology of event-related potential (ERP; Price et al., 2015). The behavioral and ERP results could complement and validate each other. The indices of attentional orientation are correlated with early ERP components such as P1, N1, and N2 (N2pc). The indices of attentional disengagement are correlated with late ERP components such as P300 and LPC. Recently, three studies combined dot-probe tasks and ERP to investigate the attentional bias toward emotional words. Shushakova et al. (2018) explored the attentional bias of individuals with Attention-Deficit/Hyperactivity Disorder (ADHD) and healthy controls. There were negative-neutral, positive-neutral, and neutral-neutral word pairs in the dot-probe task. Behaviorally, no significant effects were found. Neurophysiologically, word-locked N2pc amplitudes suggested a noteworthy attentional bias toward emotional stimuli in both groups of participants. In addition, healthy participants showed an attentional bias toward positive words. Zhang et al. (2018) investigated the attentional bias toward test-related threatening words in individuals with high or low test anxiety who were going to attend the final exams. Compared to participants with low test anxiety, those with high test anxiety showed an attentional bias toward test-related threatening words with higher attentional bias scores and enhanced N2 amplitudes. The results showed relatively amplified LPC amplitudes in participants with low test anxiety than in participants with high test anxiety. Similarly, van Heck et al. (2017) found that distinct attentional biases toward emotional words exist in healthy individuals.

Previous studies have also evidenced an attentional bias toward emotional stimuli over neutral ones (Hermans et al., 2001; Pessoa et al., 2002; Vuilleumier, 2005; Schindler and Kissler, 2016). However, the arousal of emotional pictures, faces, and words was different from each other (Keil, 2006; Carretié et al., 2008; Liu et al., 2010). Some studies have explored the attentional bias toward emotional pictures or faces (Kappenman et al., 2015; Furtak et al., 2020), while few studies have examined the attentional bias toward positive and negative words.

Until now, some issues have remained unresolved. First, various studies have investigated the dissociation between emotion-label and emotion-laden words in Indo-European languages. Little is understood about the attentional bias toward Chinese emotion-label and emotion-laden words. As the Chinese language differs greatly from Indo-European languages in terms of writing and ideographic systems, examining the attentional bias toward Chinese emotion-label and emotion-laden words could not only contribute to the comprehension of the underlying neurocognitive mechanisms of emotion-label and emotion-laden words but also help to reveal the processing mechanisms of Chinese languages. Second, valence is a crucial dimension of emotional words, and it is thus of vital significance to explore its effect on the attentional bias toward emotion-label and emotion-laden words. Therefore, the current study attempted to use dot-probe tasks with ERP technology to investigate individuals’ attentional bias toward positive emotion-label, positive emotion-laden, negative emotion-label, and negative emotion-laden words. Based on the findings of previous studies, we hypothesized that positive words would be associated with higher attentional bias scores and elicit enhanced early neural activation than negative words and that emotion-laden words would be in linkage to higher attentional bias scores and produce larger early neural activation than emotion-label words. In addition, it is hypothesized that there might be an interaction between valence (positive and negative) and emotional word type (emotion-label and emotion-laden words) and that participants might have difficulty disengaging their attention from positive emotion-laden words.

## Materials and methods

### Participants

A total of 36 (18 males) native Chinese speakers aged 18–26 years (*M* = 20.05, *SD* = 2.13) voluntarily participated in the experiment. All participants were confirmed to be right-handed using the Edinburgh Handedness Inventory (Oldfield, 1971). They had normal or corrected-to-normal vision with no history of neurological or psychiatric illness.

### Materials

To form a pool of emotional and neutral words, we selected 349 two-character Chinese words from the SUBTLEX-CH corpus (Cai and Brysbaert, 2010). There are two ways to sort emotion-label and emotion-laden words. One is the continuous ratings of the scale of the prototypicality of a word to refer to emotion (Pérez-Sánchez et al., 2021) and the other is yes/no voting for the emotional word type (Wang et al., 2019). The latter method could better meet our need for a factorial design. We followed the rating method of Wang et al. (2019) and recruited 30 raters who were homogeneous to our experimental participants. They were required to categorize the words according to the definitions of emotion-label, emotion-laden, and neutral words. If more than 80% of the raters voted for a specific emotional word type, then the word type would be determined. Through the rating, 54 positive emotion-label words, 60 positive emotion-laden words, 72 negative emotion-label words, 57 negative emotion-laden words, and 62 neutral words were identified. The pool was enlarged to 390 words after being merged with the stimuli of Wang et al. (2019), with 69 positive emotion-label words, 86 positive emotion-laden words, 87 negative emotion-label words, 86 negative emotion-laden words, and 62 neutral words. Given the fact that more neutral words were needed in the dot-probe task, we subsequently added 240 additional neutral words. All words were rated for their arousal, pleasantness, and abstractness. The frequency of words was obtained from the SUBTLEX-CH corpus (Cai and Brysbaert, 2010).

From the pool, we selected 136 emotional-neutral word pairs (34 for each type of emotional word, i.e., positive emotion-label, positive emotion-laden, negative emotion-label, and negative emotion-laden) and 68 neutral-neutral word pairs (hereinafter referred to as the “neutral word pairs”). In order to match the frequency, strokes, abstractness, arousal, and pleasantness of each word pair, one pair of each emotional word type and two neutral word pairs were deleted. Hence, there were 33 emotional-neutral pairs for each type of emotional word and 66 neutral word pairs. Within each pair, the two groups of words were matched on frequency, strokes, and abstractness (*p*s > 0.05); moreover, the pleasantness and arousal were different between emotional and neutral words (*p*s < 0.05). There was no significant difference among the arousal of positive emotion-label, positive emotion-laden, negative emotion-label, and negative emotion-laden words (*p*s > 0.05). The pleasantness of positive emotion-label words and positive emotion-laden words was significantly higher than that of neutral words (*p*s < 0.05), followed by negative emotion-label words and negative emotion-laden words (*p*s < 0.05). The difference between the pleasantness of positive emotion-label and positive emotion-laden words was non-significant, as was the pleasantness of negative emotion-label and negative emotion-laden words (*p*s > 0.05). The arousal, pleasantness, abstractness, strokes, and frequency of all neutral words were 3.27 (0.53), 4.18 (0.43), 3.74 (0.61), 17.75 (3.57), and 21.51 (28.43). The descriptive statistics of positive words and the first pair of neutral words are presented in Table 1A and those of negative words and the second pair of neutral words are shown in Table 1B. The statistical results of the properties of the experimental materials and emotional words used are presented in the Supplementary material.

**Table 1A:** The descriptive statistics of positive and neutral word pairs.

	P E-label word pair		P E-laden word pair		Neutral word pair 1	
	P E-label	Neutral	P E-laden	Neutral	Neutral 1	Neutral 2
Arousal	4.67 (0.42)	3.31 (0.59)	4.48 (0.43)	3.28 (0.51)	3.26 (0.48)	3.20 (0.63)
Pleasantness	5.61 (0.46)	4.16 (0.37)	5.59 (0.36)	4.17 (0.51)	4.22 (0.44)	4.08 (0.40)
Abstractness	3.93 (0.42)	3.76 (0.64)	3.97 (0.48)	3.93 (0.72)	3.85 (0.60)	3.72 (0.53)
Stokes	18.27 (3.20)	17.58 (4.12)	18.30 (4.30)	17.94 (3.96)	17.94 (3.12)	17.91 (3.86)
Frequency	11.75 (20.55)	18.94 (21.09)	18.77 (29.82)	25.55 (37.75)	12.77 (12.05)	19.28 (23.25)

Standard deviations are presented in parentheses. P, positive; E-label, emotion-label; E-laden, emotion-laden.

**Table 1B:** The descriptive statistics of negative and neutral word pairs.

	N E-label word pair		N E-laden word pair		Neutral word pair 2	
	N E-label	Neutral	N E-laden	Neutral	Neutral 3	Neutral 4
Arousal	4.63 (0.28)	3.26 (0.49)	4.64 (0.36)	3.17 (0.49)	3.29 (0.50)	3.42 (0.57)
Pleasantness	2.26 (0.42)	4.15 (0.43)	2.15 (0.43)	4.23 (0.44)	4.14 (0.35)	4.30 (0.49)
Abstractness	3.84 (0.45)	3.63 (0.63)	3.79 (0.55)	3.59 (0.57)	3.66 (0.62)	3.73 (0.56)
Stokes	18.58 (3.78)	17.21 (3.29)	18.06 (4.05)	18.00 (3.87)	17.94 (3.46)	17.45 (2.99)
Frequency	13.44 (29.54)	27.99 (36.69)	16.87 (33.22)	15.94 (16.61)	22.47 (29.87)	29.15 (36.43)

Standard deviations are presented in parentheses. N, negative; E-label, emotion-label; E-laden, emotion-laden.

### Procedure

**Figure 1:**
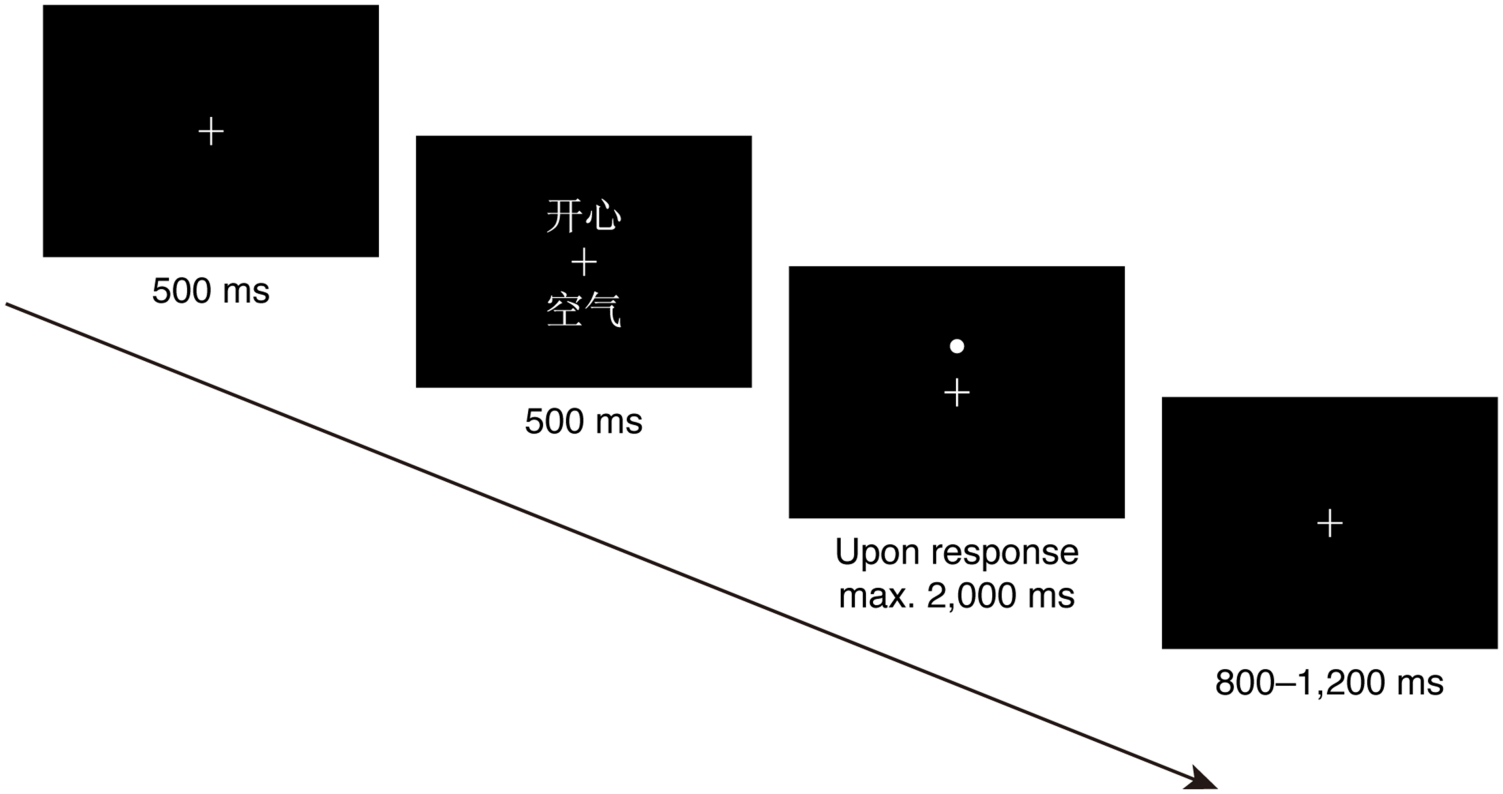
An overview of a trial in the dot-probe task.

The participants were tested in a sound-attenuated and electromagnetically shielded ERP laboratory. The experiment began with 20 practice trials whose materials were different from those in the formal experiment. Each trial began with a fixation “+” lasting 500 ms, followed by two words appearing at the upper and lower location of the fixation. The word pair (i.e., cue) was displayed for 500 ms. Immediately following the cue, the dot (i.e., target) substituted the neutral word as often as the emotional word and appeared at the upper or lower places with equal probability. A random blank (800–1,200 ms) was displayed until a response was made or until 2,000 ms had elapsed. The participants were required to judge the location of the dot as quickly and accurately as possible by pressing one of the two buttons specified in advance. The illustration of a trial is presented in Figure 1.

Each word was presented twice (once in each place), and there were four blocks, including two positive blocks and two negative ones. The blocks were presented in the order of either negative–positive–negative–positive or positive–negative–positive–negative. The positive blocks included three filler trials, 34 positive emotion-label and neutral word pairs, 34 positive emotion-laden and neutral word pairs, and 34 neutral-neutral word pairs. The negative blocks consisted of three filler trials, 34 negative emotion-label and neutral word pairs, 34 negative emotion-laden and neutral word pairs, and 34 neutral-neutral word pairs. The first three trials of each block were fillers and the order of the remaining trials was randomized. There were customized rests between blocks.

### EEG recording and analysis

Electrophysiological data were collected by 64-channel Curry 8.0 software (Neuroscan, Inc.). The Ag/AgCl electrodes were arranged based on a 10–20 system. The electrooculogram was recorded with active electrodes placed above and below the right eye as well as at the outer canthi of each eye. The online reference was the tip of the nose. The impedance was beneath 5 kΩ. The continuing electrophysiological data were recorded and the sample rate was 1,000 Hz.

The electrophysiological data were analyzed by Curry 8.0 software and re-referenced to an average reference (FP1, FP2, FPz, AF3, AF4, F1, F2, F3, F4, F5, F6, F7, F8, Fz, FC1, FC2, FC3, FC4, FC5, FC6, FCz, FT7, FT8, C1, C2, C3, C4, C5, C6, Cz, T7, T8, CP1, CP2, CP3, CP4, CP5, CP6, CPz, TP7, TP8, P1, P2, P3, P4, P5, P6, P7, P8, Pz, PO3, PO4, PO5, PO6, PO7, PO8, POz, O1, O2, Oz). Notably, prior studies examining the processing of emotion-label and emotion-laden words used an average reference (Wang et al., 2019; Wu and Zhang, 2019; Zhang et al., 2019a, 2020). To ensure the comparability of the results between our findings and previous studies, an average reference was adopted, and the bad channels were interpolated. The baseline correction was defined as −200 to 0 ms before the cue. The data were filtered between 0.1 and 30 Hz. Artifacts produced by eye blinks and movements were corrected or rejected. Brain waves smaller than −100 μV or larger than 100 μV were cast out. There were two events (i.e., the cue and the dot) in this experiment and their epochs were as follows. For the cue, −200 to 500 ms upon the appearance of the cue and −200 to 0 ms was the baseline. For the dot, 0 to 500 ms upon the appearance of the target and the baseline was −200 to 0 ms before the cue in accordance with preceding studies (e.g., Poulsen et al., 2005; Zhong et al., 2011; Pintzinger et al., 2017; Crago et al., 2019).

Based on prior studies (e.g., van Heck et al., 2017; Shushakova et al., 2018) and visual inspection of the grand averages, the extracted ERP data of the cues were analyzed for the following three time windows: 120–150 ms (P1), 185–240 ms (N2) in the temporoparieto-occipital region and 190–220 ms (P2) in the fronto-central region. The extracted ERP data for the target was analyzed for the following three time windows: 620–660 ms (i.e., 120–160 ms, P1), 700–760 ms (i.e., 200–260 ms, N2), and 860–930 ms (i.e., 360–430 ms, P300). The time windows and selected electrodes are presented in Table 2.

**Table 2:** The time windows and selected electrodes for ERP components of the cue and the target.

	Component	Time window (ms)	Electrodes
Cue	P1	120–150	PO5, PO7; PO6, PO8
	N2	185–240	P7, PO7; P8, PO8
	P2	190–220	C1, C2, Cz, FC1, FC2, FCz
Target	P1	620–660 (120–160)	PO5, PO7; PO6, PO8
	N2	700–760 (200–260)	PO5, PO7; PO6, PO8
	P300	860–930 (360–430)	CP1, CP2, CPz, P1, P2, Pz

## Results

The data of 6 participants were excluded from further analysis due to artifacts (more than 20% of the trials), leaving 30 participants (13 males) aged 18–26 years (*M* = 19.73, *SD* = 2.12). Trials with wrong responses and RTs more than 3 *SD* were eliminated (2.15%). The accuracy of the participants ranged from 97.47 to 100% (*M* = 99.34, *SD* = 0.57). For behavioral data, RTs to the dots were analyzed to investigate the attentional bias toward emotion-label and emotion-laden words. For electrophysiological data, only the targets with correct responses (94.52%) and cues (95.85%) were analyzed.

### Behavioral results

The indices of attentional bias, orientation, and disengagement were calculated. These indices were tested against 0 via one-sample *t* tests and mean RTs were analyzed using analyses of variance (ANOVAs) with the within-subject factors valence (positive, negative) and emotional word type (emotion-label, emotion-laden). The means of the indices of attentional bias, orientation, and disengagement as well as their standard deviations are shown in Table 3.

**Table 3:** The descriptive data of attentional bias, orientation, and disengagement (ms).

	Negative emotion-label words	Negative emotion-laden words	Positive emotion-label words	Positive emotion-laden words
Bias	2.70 (14.17)	−2.03 (11.15)	−4.23 (14.17)	3.55 (12.67)
Orientation	−0.86 (11.02)	−1.91 (9.56)	−4.05 (16.08)	−1.26 (11.46)
Disengagement	3.56 (12.17)	−0.12 (9.69)	−0.19 (10.80)	4.81 (12.14)

Standard deviations are presented in parentheses.

#### Attentional bias

One-sample *t* tests of the attentional bias indices toward positive emotion-label, positive emotion-laden, negative emotion-label, and negative emotion-laden words showed that there was no significant difference between the attentional bias indices of the four types of emotional words and 0 (*p*s ≥ 0.113). The repeated measures ANOVA of attentional bias scores revealed that the main effects of valence and emotional word type were not significant, *F*(1, 29) = 0.077, *p* = 0.784, *F*(1, 29) = 0.495, *p* = 0.487. The interaction effect between emotional word type and valence was significant, *F*(1, 29) = 10.714, *p* = 0.003, η_p_^2^ = 0.270. Simple effect analysis found an attentional bias away from positive emotion-label words (−4.23 ms) and toward positive emotion-laden words (3.55 ms), *F*(1, 29) = 7.493, *p* = 0.010. In addition, an attentional bias toward negative emotion-label words (2.70 ms) and away from positive emotion-label words (−4.23 ms) was observed, *F*(1, 29) = 6.119, *p* = 0.019.

#### Attentional orientation

One-sample *t* tests of the attentional orientation indices toward positive emotion-label, positive emotion-laden, negative emotion-label, and negative emotion-laden words showed that there was no significant difference between the attentional orientation indices of the four types of emotional words and 0 (*p*s ≥ 0.179). The repeated measures ANOVA of attentional orientation indices revealed no significant main effects or interaction effects (*F*s ≤ 1.240, *p*s ≥ 0.275).

#### Attentional disengagement

One-sample *t* tests of the attentional disengagement indices revealed a significant difference between the attentional disengagement index of positive emotion-laden words (4.81 ms) and 0, *t*(29) = 2.170, *p* = 0.038, indicating that individuals had difficulty shifting attention away from positive emotion-laden words. There was no significant difference between the attentional disengagement indices of positive emotion-label, negative emotion-label, and negative emotion-laden words and 0 (*p*s ≥ 0.120). The results of the repeated measures ANOVA of the attentional disengagement indices revealed that the main effects of valence and emotional word type were not significant, *F*(1, 29) = 0.076, *p* = 0.785, *F*(1, 29) = 0.149, *p* = 0.702. The interaction effect between emotional word type and valence was significant, *F*(1, 29) = 9.465, *p* = 0.005, η_p_^2^ = 0.246. The simple effect analysis showed that it was more difficult for individuals to disengage their attention from positive emotion-laden words (4.81 ms) as compared to positive emotion-label ones (−0.19 ms), *F*(1, 29) = 4.580, *p* = 0.041. This emotional word type effect was marginally significant during negative word processing, *F*(1, 29) = 3.109, *p* = 0.088. It was more difficult for individuals to disengage attention from emotion-label words (3.56 ms) relative to emotion-laden ones (−0.12 ms). Additionally, the valence effect was marginally significant during emotion-laden word processing, *F*(1, 29) = 3.365, *p* = 0.077. Specifically, it was more difficult for individuals to disengage attention from positive emotion-laden words (4.81 ms) relative to negative ones (−0.12 ms).

To summarize the behavioral results, an attentional bias toward positive emotion-laden words rather than positive emotion-label ones was observed, and this bias arose from the difficulty in disengaging attention from positive emotion-laden words. Furthermore, an attentional bias toward negative emotion-label words as compared with positive emotion-label words was found.

### ERP results

The hemisphere effect could affect the amplitudes of P1 (e.g., Herbert et al., 2008; Kissler et al., 2009; Frühholz et al., 2011; Zhang et al., 2017) and N2 (e.g., Zhang et al., 2018; Wu and Zhang, 2019) elicited by emotional words. Hence, for the analysis of the mean amplitudes of P1 and N2 elicited by the cues, repeated measures ANOVAs were conducted separately with hemisphere (left and right), valence (positive and negative), and emotional word type (emotion-label words and emotion-laden words) as within-subject factors. P2 was examined in a sole channel group.

For the analysis of the mean amplitudes of P1 and N2 produced by the target, repeated measures ANOVAs were conducted separately with hemisphere (left and right), congruency (congruent and incongruent), valence (positive and negative), and emotional word type (emotion-label words and emotion-laden words) as within-subject factors. For the analysis of the mean amplitudes of P300 elicited by the target, a repeated measures ANOVA was conducted with congruency (congruent, incongruent), valence (positive and negative), and emotional word type (emotion-label words and emotion-laden words) as within-subject factors.

A *p* < 0.05 was considered significant and was corrected through the Greenhouse–Geisser epsilon when needed. For pairwise comparisons, alpha levels were Bonferroni adjusted. Only significant effects were provided with figures of brain wave and topography.

#### The cue

##### P1

The main effect of hemisphere was significant, *F*(1, 29) = 5.251, *p* = 0.029, η_p_^2^ = 0.153. The word pairs elicited a larger P1 in the right hemisphere (2.72 μV) than in the left hemisphere (1.54 μV).

The interaction effect between valence and hemisphere was significant, *F*(1, 29) = 5.551, *p* = 0.025, η_p_^2^ = 0.161. Simple effect analysis showed that both positive and negative word pairs elicited larger P1 in the right hemisphere than in the left hemisphere, *F*(1, 29) = 7.269, *p* = 0.012, *F*(1, 29) = 3.566, *p* = 0.069. The valence effect was not significant in either the left or the right hemispheres (*F*s ≤ 2.434, *p*s ≥ 0.130).

The interaction effect between emotional word type, valence, and hemisphere was significant, *F*(1, 29) = 4.311, *p* = 0.047, η_p_^2^ = 0.129. To further investigate the neurocognitive mechanisms of emotion-label and emotion-laden words, two repeated measures ANOVA with valence and hemisphere as within-subject factors were conducted on the amplitudes elicited by emotion-label and emotion-laden word pairs.

The analysis of the amplitudes elicited by emotion-label word pairs showed that only hemisphere effect was significant, *F*(1, 29) = 5.000, *p* = 0.033, η_p_^2^ = 0.147, emotion-label word pairs elicited larger P1 in the right hemisphere (2.70 μV) than in the left hemisphere (1.51 μV). The main effect of valence and the interaction effect between valence and hemisphere were not significant, *F*(1, 29) = 1.242, *p* = 0.274, *F*(1, 29) = 0.087, *p* = 0.770.

The analysis of the amplitudes produced by emotion-laden word pairs manifested that the main effect of hemisphere was significant, *F*(1, 29) = 5.101, *p* = 0.032, η_p_^2^ = 0.150, emotion-laden word pairs elicited amplified P1 in the right hemisphere (2.73 μV) than in the left hemisphere (1.57 μV). The interaction effect between valence and hemisphere was significant, *F*(1, 29) = 7.955, *p* = 0.009, η_p_^2^ = 0.215. The simple effect analysis showed that positive emotion-laden word pairs produced enhanced P1 in the right hemisphere (2.95 μV) than in the left hemisphere (1.44 μV), *F*(1, 29) = 8.922, *p* = 0.006. The hemisphere effect was not significant during negative word processing, *F*(1, 29) = 2.193, *p* = 0.149.

**Figure 2:**
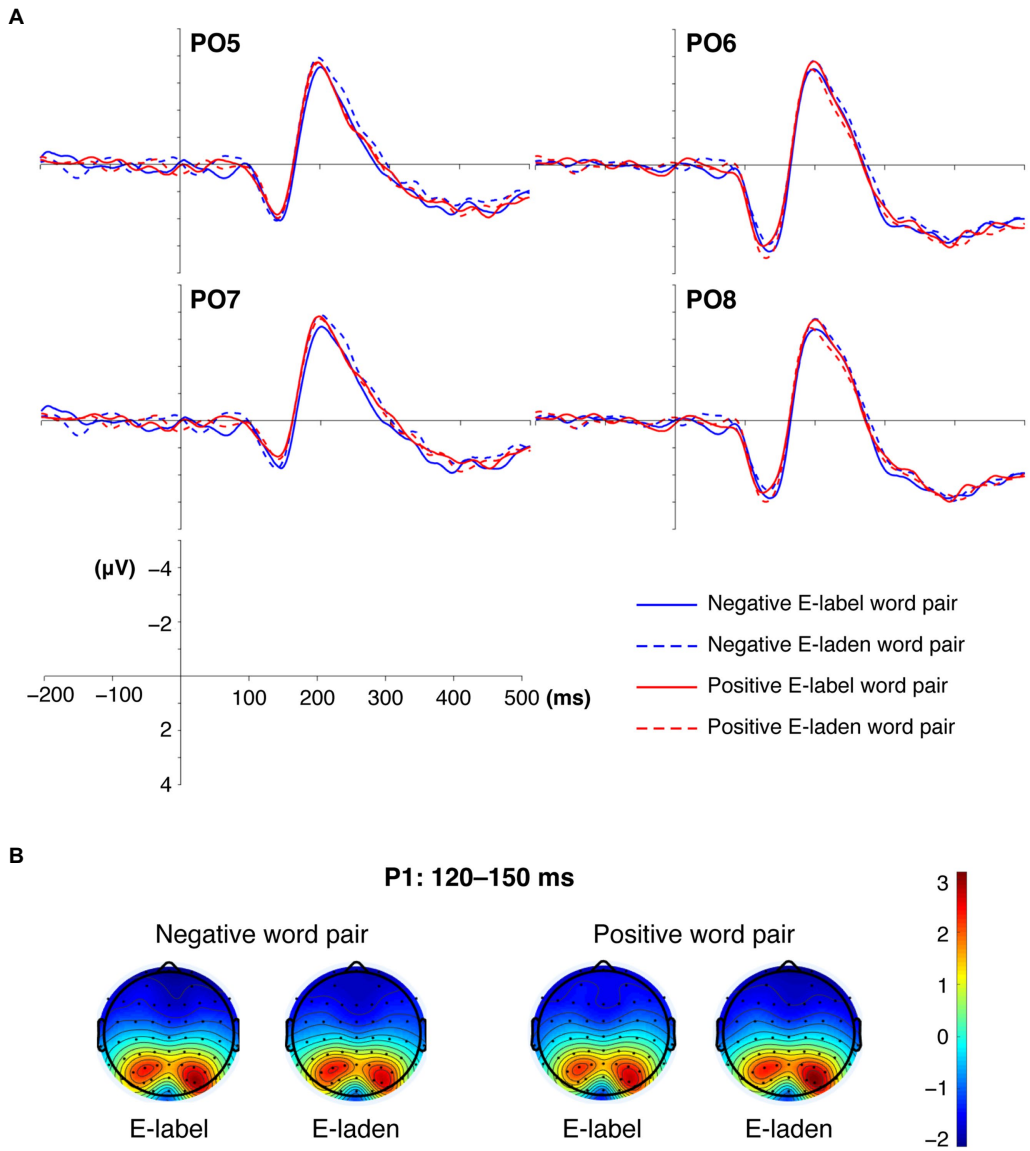
Mean grand-average ERPs **(A)** and the topography of cortical responses **(B)** to the four types of emotional word pairs over parieto-occipital sites for P1. E-label, emotion-label; E-laden, emotion-laden.

No other significant main effects or interaction effects were observed (*F*s ≤ 1.172, *p*s ≥ 0.288). Mean grand-average brain waves and topography of P1 are displayed in Figure 2.

##### N2

No significant main effects or interaction effects were found (*F*s ≤ 2.836, *p*s ≥ 0.103).

##### P2

No significant main effects or interaction effects were found (*F*s ≤ 0.498, *p*s ≥ 0.486).

The ERP results of the cue in the experiment are shown in Table 4.

**Table 4:** The ERP results of the cue in the experiment.

ERPs	Effects	*F*(1, 29)	*p*	η_p_^2^
P1	Emotional word type	0.071	0.792	0.002
	Valence	0.416	0.524	0.014
	Hemisphere	5.251	0.029*	0.153
	Emotional word type × Valence	1.172	0.288	0.039
	Emotional word type × Hemisphere	0.020	0.888	0.001
	Valence × Hemisphere	5.551	0.025*	0.161
	Emotional word type × Valence × Hemisphere	4.311	0.047*	0.129
N2	Emotional word type	0.760	0.390	0.026
	Valence	0.346	0.561	0.012
	Hemisphere	0.086	0.771	0.003
	Emotional word type × Valence	2.836	0.103	0.089
	Emotional word type × Hemisphere	1.269	0.269	0.042
	Valence × Hemisphere	0.458	0.504	0.016
	Emotional word type × Valence × Hemisphere	0.051	0.823	0.002
P2	Emotional word type	0.498	0.486	0.017
	Valence	0.077	0.783	0.003
	Emotional word type × Valence	0.389	0.538	0.013

*p < 0.05.

#### The target

##### P1

The congruency effect was marginally significant, *F*(1, 29) = 2.914, *p* = 0.098, η_p_^2^ = 0.091. The dots under congruent conditions (1.31 μV) elicited an enhanced P1 than those under incongruent conditions (1.02 μV). The main effect of hemisphere was marginally significant, *F*(1, 29) = 3.429, *p* = 0.074, η_p_^2^ = 0.106, and the dots produced larger P1 in the right hemisphere (1.47 μV) than in the left hemisphere (0.85 μV).

The interaction effect between valence and congruency was marginally significant, *F*(1, 29) = 3.350, *p* = 0.078, η_p_^2^ = 0.104. The simple effect analysis revealed that the dots following negative words under congruent conditions (1.39 μV) elicited enhanced P1 than those under incongruent conditions (0.82 μV), *F*(1, 29) = 7.792, *p* = 0.009.

No other significant main effects or interaction effects were found (*F*s ≤ 2.829, *p*s ≥ 0.103).

##### N2

The interaction effect between congruency and hemisphere was significant, *F*(1, 29) = 4.757, *p* = 0.037, η_p_^2^ = 0.141. The simple effect analysis showed that the dots under incongruent conditions (−0.94 μV) elicited larger N2 than those under congruent conditions (−0.57 μV), *F*(1, 29) = 6.457, *p* = 0.017.

**Figure 3:**
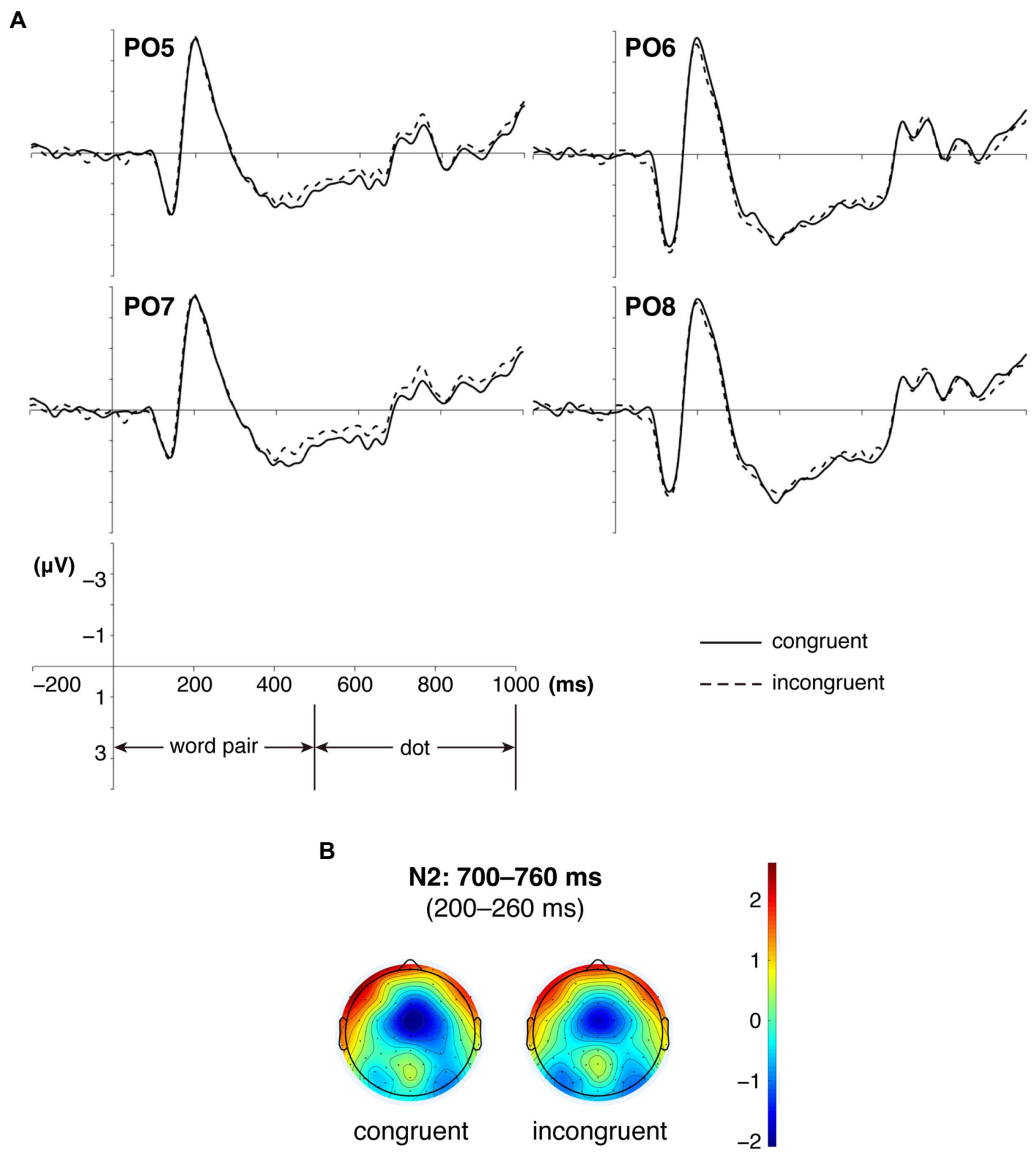
Mean grand-average ERPs **(A)** and the topography of cortical responses **(B)** to the dots under congruent and incongruent conditions over fronto-central and central sites for N2.

No other significant main effects or interaction effects were observed (*F*s ≤ 2.526, *p*s ≥ 0.123). Mean grand-average brain waves and topography of N2 are displayed in Figure 3.

##### P300

The main effect of emotional word type was marginally significant, *F*(1, 29) = 3.406, *p* = 0.075, η_p_^2^ = 0.105, the dots following emotion-laden words (3.07 μV) eliciting larger P300 than those following emotion-label words (2.89 μV). Other main effects or interaction effects were not significant (*F*s ≤ 1.904, *p*s ≥ 0.178).

The ERP results of the target in the experiment are shown in Table 5.

In summary, the ERP results revealed an early attentional bias toward positive emotion-laden words (P1 of the cue) and an attentional bias toward emotional words instead of neutral ones (N2 of the target).

**Table 5:** The ERP results of the target in the experiment.

ERPs	Effects	*F*(1, 29)	*p*	η_p_^2^
P1	Emotional word type	0.705	0.408	0.024
	Valence	0.335	0.567	0.011
	Congruency	2.914	0.098	0.091
	Hemisphere	3.429	0.074	0.106
	Emotional word type × Valence	2.829	0.103	0.089
	Emotional word type × Congruency	0.025	0.876	0.001
	Emotional word type × Hemisphere	0.077	0.784	0.003
	Congruency × Valence	3.350	0.078	0.104
	Congruency × Hemisphere	1.588	0.218	0.052
	Valence × Hemisphere	0.276	0.603	0.009
	Congruency × Valence × Hemisphere	0.303	0.586	0.010
	Congruency × Valence × Emotional word type	0.003	0.956	0.000
	Congruency × Emotional word type × Hemisphere	2.309	0.139	0.074
	Emotional word type × Valence × Hemisphere	0.301	0.587	0.010
	Emotional word type × Valence × Congruency × Hemisphere	0.010	0.921	0.000
N2	Emotional word type	1.425	0.242	0.047
	Valence	0.070	0.793	0.002
	Congruency	2.526	0.123	0.080
	Hemisphere	0.171	0.683	0.006
	Emotional word type × Valence	2.065	0.161	0.066
	Congruency × Valence	3.552	0.070	0.109
	Congruency × Emotional word type	0.537	0.469	0.018
	Valence × Hemisphere	0.023	0.880	0.001
	Emotional word type × Hemisphere	0.132	0.719	0.005
	Congruency × Hemisphere	4.757	0.037*	0.141
	Emotional word type × Valence × Congruency	1.134	0.296	0.038
	Congruency× Valence × Hemisphere	1.500	0.231	0.049
	Congruency × Emotional word type × Hemisphere	1.111	0.301	0.037
	Emotional word type × Valence × Hemisphere	0.878	0.357	0.029
	Emotional word type × Valence × Congruency × Hemisphere	2.232	0.146	0.071
P300	Emotional word type	3.406	0.075	0.105
	Valence	1.071	0.309	0.036
	Congruency	0.199	0.659	0.007
	Emotional word type × Valence	0.115	0.737	0.004
	Congruency × Valence	0.259	0.615	0.009
	Congruency × Emotional word type	1.904	0.178	0.062
	Emotional word type × Valence × Congruency	0.090	0.766	0.003

*p < 0.05.

## Discussion

The current study investigated the valence effect on the attentional bias toward emotion-label and emotion-laden words, yielding three main findings. Firstly, an attentional bias toward positive emotion-laden words was found and the behavioral result was evidenced by the electrophysiological finding that positive emotion-laden words elicited an enhanced P1 in the right hemisphere than in the left hemisphere. Secondly, an attentional bias toward negative emotion-label words rather than positive emotion-label words was observed. Thirdly, the dots eliciting an enhanced N2 under incongruent conditions than the congruent conditions reflected that emotional words automatically captured more attentional resources than neutral ones.

Notably, individuals had difficulty disengaging attention from positive emotion-laden words rather than positive emotion-label ones resulting in an attentional bias toward positive emotion-laden words. Consistent with the behavioral data, the ERP data also evidenced an early attentional bias toward positive emotion-laden words. There have thus far been few studies investigating the valence effect on the attentional bias toward different types of emotional words. The finding that emotion-laden words attracting more attentional resources than emotion-label ones was consistent with preceding studies (Altarriba and Basnight-Brown, 2010; Kazanas and Altarriba, 2015, 2016a,b; El-Dakhs and Altarriba, 2019; Bromberek-Dyzman et al., 2021), which demonstrated that longer RTs were associated with emotion-laden words. There were two reasons for this phenomenon. For one thing, the conceptual meanings of emotion-label words were the same as their affective meanings, which could be accessed directly, whereas the affective meanings of emotion-laden words were different from their conceptual ones. The conceptual meanings of emotion-laden words have to be accessed first and then the affective meanings can become accessible. This “mediated” process (Altarriba and Basnight-Brown, 2010) would cost more attentional resources. For another, emotion-label words refer to emotions directly, and people experienced emotions almost every day. That is to say, compared to emotion-laden words, emotion-label ones were associated with stronger embodiment and their processing could directly access the sensorimotor and neural mechanisms related to emotions (Niedenthal, 2007; Barsalou, 2008; Horchak et al., 2014). Therefore, an attentional bias toward emotion-laden words was observed during positive word processing.

The electrophysiological data of the cues showed that emotional word type, valence, and hemisphere interactively affect the P1 amplitudes elicited by emotional word pairs. When participants processed emotion-label word pairs, there was no significant difference between the mean amplitudes of negative and positive word pairs in either the right or the left hemispheres. When the participants processed positive emotion-laden word pairs, the word pairs elicited larger P1 in the right hemisphere than in the left hemisphere. In emotion perception, the right hemisphere had an advantage over the left hemisphere (Borod et al., 1998; Smith and Bulman-Fleming, 2005; Zhang et al., 2017). Emotion-laden words corresponded to at least one kind of emotion, while emotion-label words only referred to a specific kind of emotion. Hence, a significant hemisphere effect was found during emotion-laden word processing. These findings indicated that in the early perceptual processing stage, individuals paid more attention to positive emotion-laden words and showed an attentional bias toward positive emotion-laden words. This finding is similar to previous studies demonstrating an early attentional orientation toward positive stimuli (Carretié et al., 2004; Zhong et al., 2011; Shushakova et al., 2018), indicating the automatic evaluation of the emotional contents of stimuli (Hermans et al., 2001; Pessoa et al., 2002; Vuilleumier, 2005; Kissler et al., 2009; Schindler and Kissler, 2016). For instance, Shushakova et al. (2018) found in a dot-probe task that positive-neutral word pairs other than negative-neutral word pairs elicited larger N2pc. Some other studies reported that positive and neutral pictures could produce enhanced N2 than negative ones (Carretié et al., 2004), suggesting that healthy individuals showed an attentional bias toward positive stimuli. Zhong et al. (2011) also reported that healthy individuals showed an attentional bias toward positive pictures, and positive pictures in congruent conditions elicited enhanced P1 than those in incongruent conditions. Pool et al. (2016) pointed out that P1 and N2pc evoked by cues indicated an early attentional orientation. Thus, in our study, positive emotion-laden word pairs elicited significantly larger P1 indicating an early attentional orientation toward positive emotion-laden words.

The finding that individuals showed an attentional bias toward negative emotion-label words relative to positive emotion-label words was consistent with that of a preceding study (Sutton and Altarriba, 2011), which was the only existing study examining the attentional bias toward positive emotion-label and negative emotion-label words. The results of the dot-probe task in Sutton and Altarriba (2011) showed that the RTs to the dots following negative emotion-label words were much shorter than those following neutral words; in comparison, such an effect was not found during positive emotion-label word processing. The results indicated that negative emotion-label words could automatically capture individuals’ attention. Experiment 1 in Knickerbocker and Altarriba (2013) used RSVP tasks to explore the processing of negative emotion-label and negative emotion-laden words. They found that in the early processing stage of words, individuals showed attentional bias toward negative emotion-label words. These findings were supportive of the argument of Ho et al. (2016) that when there was a competition between two stimuli, negative stimuli would capture attention earlier than positive or neutral ones and attract enhanced perception. An attentional bias toward negative emotion-label words instead of positive emotion-label ones was not observed in the ERP results, which was consistent with the findings of Zhang et al. (2018). In their study, individuals with high test anxiety showed attentional vigilance to threatening words related to tests, and individuals with low test anxiety showed attentional avoidance of threatening words related to tests. However, individuals showed no attentional bias toward threatening words that were unrelated to tests. In our study, the materials were general negative words that had little correlation with the states of the participants. Therefore, our findings were similar to the results of Zhang et al. (2018). Some other studies, however, found an attentional bias toward negative pictures (Kappenman et al., 2015; Furtak et al., 2020). Generally speaking, individuals showed a negative bias toward the emotional contents of pictures or faces and a positive bias toward the emotional information of words (Bayer and Schacht, 2014; Yuan et al., 2019). This might be because pictures or faces had higher arousal than words (Keil, 2006; Carretié et al., 2008; Liu et al., 2010). In particular, high arousal of stimuli could activate the defensive motivation system, leading to a negative bias; conversely, low arousal of stimuli could activate the appetitive motivation system, leading to a positive bias (Liu et al., 2010; Yuan et al., 2019). The above findings demonstrated that there was an interaction between emotional word type and valence on the attentional bias toward emotional words—a negative bias was found during the processing of emotion-label words, supporting the automatic vigilance hypothesis (Pratto and John, 1991). The embodiment degree of emotion-laden words was weaker than that of emotion-label ones. Therefore, a bias toward emotional words was not observed during emotion-laden word processing.

The fact that dots following neutral words (incongruent conditions) producing an enhanced N2 than those following emotional words (congruent conditions) indicated that emotional words automatically captured more spatial attention than neutral ones. If emotional words could automatically capture more attention, then the attention would be allocated to the location following emotional words. When the participants had to respond to the dots following neutral words, there would be a shift of attention from the location following emotional words to the place following neutral ones. This process would require more attention. Therefore, the dots following neutral words produced a larger N2 than those following emotional ones. This finding was consistent with a number of prior studies demonstrating an attentional bias toward emotional stimuli rather than neutral ones (Vuilleumier, 2005; Yiend, 2010; Pourtois et al., 2013).

## Conclusion

To our knowledge, this study is the first attempt to explore how valence interacted with emotional word type on the attentional bias toward emotional words with the ERP technique. The behavioral data showed that there was an interaction between emotional word type and valence. Compared to positive emotion-label words, individuals had difficulty disengaging their attention from positive emotion-laden words, thereby leading to an attentional bias toward them. Individuals showed an attentional bias toward negative emotion-label words relative to positive emotion-label words. Also, the electrophysiological data showed an early attentional bias toward positive emotion-laden words. An attention bias toward emotional words rather than neutral ones was also found. According to both behavioral and electrophysiological results, the attentional bias toward emotional words is a dynamic process. In the early stages, the participants showed an attentional bias toward positive emotion-laden words. In the middle stages, the degree of attentional bias toward different types of emotional words was similar. In the final stage, individuals showed an attentional bias toward positive emotion-laden words again because of the difficulty in disengagement. Besides, an attentional bias toward negative emotion-label words was also observed in this final stage.

Although the findings of the present study reveal the cognitive mechanisms of valence and emotional word type effects on attentional bias toward emotional words, there remains a limitation to be considered in future research. The current study did not take the emotional states or trait anxiety of individuals into consideration, which might have an effect on the attentional bias toward emotional words. Future studies should recruit more participants with different levels of emotional states or different traits to further explore the effect of individual differences on attentional bias toward emotion-label and emotion-laden words.

## Data availability statement

The raw data supporting the conclusions of this article will be made available by the authors upon requirement, without undue reservation.

## Ethics statement

The studies involving human participants were reviewed and approved by the Ethics Board of Artificial Intelligence and Human Languages Lab of Beijing Foreign Studies University. The participants provided their written informed consent to participate in this study.

## Author contributions

JL: conceptualization, data collection, formal analysis, investigation, methodology, resources, visualization, writing-original draft, and writing—review and editing. LF: conceptualization, funding acquisition, investigation, methodology, project administration, resources, supervision, and writing—review and editing. JJ and XZ: data collection and writing—review and editing. CL: data collection. LT: writing—review and editing. WF: methodology. All authors contributed to the article and approved the submitted version.

## Funding

This study was supported by the First-Class Disciplines Project of Beijing Foreign Studies University (2022SYLZD008) and Tianjin Philosophical and Social Science Grant (TJYY21-014).

## Conflict of interest

The authors declare that the research was conducted in the absence of any commercial or financial relationships that could be construed as a potential conflict of interest.

## Publisher’s note

All claims expressed in this article are solely those of the authors and do not necessarily represent those of their affiliated organizations, or those of the publisher, the editors and the reviewers. Any product that may be evaluated in this article, or claim that may be made by its manufacturer, is not guaranteed or endorsed by the publisher.

## References

[ref1] Altarriba J. Basnight-Brown D. M. (2010). The representation of emotion vs. emotion-laden words in English and Spanish in the affective Simon task. Int. J. Billing. 15, 310–328. doi: 10.1177/1367006910379261

[ref2] Barsalou L. W. (2008). Grounded cognition. Annu. Rev. Psychol. 59, 617–645. doi: 10.1146/annurev.psych.59.103006.093639 17705682

[ref3] Bayer M. Schacht A. (2014). Event-related brain responses to emotional words, pictures, and faces: A cross-domain comparison. Front. Psychol. 5:1106. doi: 10.3389/fpsyg.2014.01106, PMID: 25339927 PMC4186271

[ref4] Borod J. C. Cicero B. A. Obler L. K. Welkowitz J. Erhan H. M. Santschi C.. (1998). Right hemisphere emotional perception: evidence across multiple channels. Neuropsychology 12, 446–458. doi: 10.1037/0894-4105.12.3.446, PMID: 9673999

[ref5] Bromberek-Dyzman K. Jończyk R. Vasileanu M. Niculescu-Gorpin A.-G. Bąk H. (2021). Cross-linguistic differences affect emotion and emotion-laden word processing: evidence from polish-English and Romanian-English bilinguals. Int. J. Billing. 25, 1161–1182. doi: 10.1177/1367006920987306

[ref6] Cai Q. Brysbaert M. (2010). SUBTLEX-CH: Chinese word and character frequencies based on film subtitles. PLoS One 5:e10729. doi: 10.1371/journal.pone.0010729.t001 20532192 PMC2880003

[ref7] Carretié L. Hinojosa J. A. Albert J. López-Martín S. De La Gandara B. S. Igoa J. M.. (2008). Modulation of ongoing cognitive processes by emotionally intense words. Psychophysiology 45, 188–196. doi: 10.1111/j.1469-8986.2007.00617.x, PMID: 17971056

[ref8] Carretié L. Hinojosa J. A. Martín-Loeches M. Mercado F. Tapia M. (2004). Automatic attention to emotional stimuli: Neural correlates. Hum. Brain Mapp. 22, 290–299. doi: 10.1002/hbm.20037, PMID: 15202107 PMC6871850

[ref9] Citron F. M. M. (2012). Neural correlates of written emotion word processing: A review of recent electrophysiological and hemmodynamic neuroimaging studies. Brain Lang. 122, 211–226. doi: 10.1016/j.bandl.2011.12.007, PMID: 22277309

[ref10] Conrad M. Recio G. Jacobs A. M. (2011). The time course of emotion effects in first and second language processing: A cross cultural ERP study with German–Spanish bilinguals. Front. Psychol. 2:351. doi: 10.3389/fpsyg.2011.00351, PMID: 22164150 PMC3230907

[ref11] Crago R. V. Renoult L. Biggart L. Nobes G. Satmarean T. Bowler J. O. (2019). Physical aggression and attentional bias to angry faces: an event related potential study. Brain Res. 1723:146387. doi: 10.1016/j.brainres.2019.146387, PMID: 31419430

[ref12] Dolan R. J. (2002). Emotion, cognition, and behavior. Science 298, 1191–1194. doi: 10.1126/science.1076358 12424363

[ref13] El-Dakhs D. A. S. Altarriba J. (2019). How do emotion word type and valence influence language processing? The case of Arabic-English bilinguals. J. Psycholinguist. Res. 48, 1063–1085. doi: 10.1007/s10936-019-09647-w, PMID: 31089949

[ref14] Fan L. Xu Q. Wang X. Xu F. Yang Y. Lu Z. (2018). The automatic activation of emotion words measured using the emotional face-word Stroop task in late Chinese-English bilinguals. Cognit. Emot. 32, 315–324. doi: 10.1080/02699931.2017.1303451, PMID: 28332423

[ref15] Frühholz S. Jellinghaus A. Herrmann M. (2011). Time course of implicit processing and explicit processing of emotional faces and emotional words. Biol. Psychol. 87, 265–274. doi: 10.1016/j.biopsycho.2011.03.008, PMID: 21440031

[ref16] Furtak M. Doradzinska L. Ptashynska A. Mudrik L. Nowicka A. Bola M. (2020). Automatic attention capture by threatening, but not by semantically incongruent natural scene images. Cereb. Cortex 30, 4158–4168. doi: 10.1093/cercor/bhaa040 32198506

[ref17] Herbert C. Junghofer M. Kissler J. (2008). Event related potentials to emotional adjectives during reading. Psychophysiology 45, 487–498. doi: 10.1111/j.1469-8986.2007.00638.x, PMID: 18221445

[ref18] Hermans D. De Houwer J. Eelen P. (2001). A time course analysis of the affective priming effect. Cognit. Emot. 15, 143–165. doi: 10.1080/02699930125768

[ref19] Hinojosa J. A. Moreno E. M. Ferré P. (2020a). Affective neurolinguistics: towards a framework for reconciling language and emotion. Lang. Cogn. Neurosci. 35, 813–839. doi: 10.1080/23273798.2019.1620957

[ref20] Ho M.-C. Li S.-H. Yeh S.-L. (2016). Early attentional bias for negative words when competition is induced. Atten. Percept. Psychophys. 78, 1030–1042. doi: 10.3758/s13414-016-1084-9, PMID: 26980328

[ref21] Horchak O. V. Giger J.-C. Cabral M. Pochwatko G. (2014). From demonstration to theory in embodied language comprehension: A review. Cogn. Syst. Res. 29-30, 66–85. doi: 10.1016/j.cogsys.2013.09.002

[ref22] Kappenman E. S. MacNamara A. Proudfit G. H. (2015). Electrocortical evidence for rapid allocation of attention to threat in the dot-probe task. Soc. Cogn. Affect. Neurosci. 10, 577–583. doi: 10.1093/scan/nsu098, PMID: 25062842 PMC4381248

[ref23] Kazanas S. Altarriba J. (2015). The automatic activation of emotion and emotion-laden words: evidence from a masked and unmasked priming paradigm. Am. J. Psychol. 128, 323–336. doi: 10.5406/amerjpsyc.128.3.0323, PMID: 26442339

[ref24] Kazanas S. Altarriba J. (2016a). Emotion word type and affective valence priming at a long stimulus onset asynchrony. Lang. Speech 59, 339–352. doi: 10.1177/0023830915590677 29924529

[ref25] Kazanas S. Altarriba J. (2016b). Emotion word processing: effects of word type and valence in Spanish-English bilinguals. J. Psycholinguist. Res. 45, 395–406. doi: 10.1007/s10936-015-9357-3, PMID: 25732384

[ref26] Keil A. (2006). Macroscopic brain dynamics during verbal and pictorial processing of affective stimuli. Prog. Brain Res. 156, 217–232. doi: 10.1016/s0079-6123(06)56011-x, PMID: 17015082

[ref27] Kissler J. (2020). Affective neurolinguistics: a new field to grow at the intersection of emotion and language? – commentary on Hinojosa et al., 2019. Lang. Cogn. Neurosci. 35, 850–857. doi: 10.1080/23273798.2019.1694159

[ref28] Kissler J. Herbert C. Winkler I. Junghofer M. (2009). Emotion and attention in visual word processing: an ERP study. Biol. Psychol. 80, 75–83. doi: 10.1016/j.biopsycho.2008.03.004, PMID: 18439739

[ref29] Knickerbocker H. Altarriba J. (2013). Differential repetition blindness with emotion and emotion-laden word types. Vis. Cogn. 21, 599–627. doi: 10.1080/13506285.2013.815297

[ref30] Koster E. H. Crombez G. Verschuere B. De Houwer J. (2004). Selective attention to threat in the dot probe paradigm: differentiating vigilance and difficulty to disengage. Behav. Res. Ther. 42, 1183–1192. doi: 10.1016/j.brat.2003.08.001, PMID: 15350857

[ref31] Liu J. Fan L. Tian L. Li C. Feng W. (2022). The neural mechanisms of explicit and implicit processing of Chinese emotion-label and emotion-laden words: evidence from emotional categorization and emotional Stroop tasks. Lang. Cogn. Neurosci. Advance online publication. doi: 10.1080/23273798.2022.2093389

[ref32] Liu J. Fan L. Yin H.-S. (2020). A bibliometric analysis on cognitive processing of emotional words. DSH 35, 353–365. doi: 10.1093/llc/fqz025

[ref33] Liu B. Jin Z. Wang Z. Hu Y. (2010). The interaction between pictures and words: evidence from positivity offset and negativity bias. Exp. Brain Res. 201, 141–153. doi: 10.1007/s00221-009-2018-8, PMID: 19779702

[ref34] MacLeod C. Mathews A. Tata P. (1986). Attentional bias in emotional disorders. J. Abnorm. Psychol. 95, 15–20. doi: 10.1037//0021-843x.95.1.15 3700842

[ref35] Martin J. M. Altarriba J. (2017). Effects of valence on hemispheric specialization for emotion word processing. Lang. Speech 60, 597–613. doi: 10.1177/0023830916686128, PMID: 29216810

[ref36] Niedenthal P. M. (2007). Embodying emotion. Science 316, 1002–1005. doi: 10.1126/science.1136930 17510358

[ref37] Oldfield R. C. (1971). The assessment and analysis of handedness: The Edinburgh inventory. Neuropsychologia 9, 97–113. doi: 10.1016/0028-3932(71)90067-4, PMID: 5146491

[ref38] Pavlenko A. (2008). Emotion and emotion-laden words in the bilingual lexicon. Biling. Lang. Cogn. 11, 147–164. doi: 10.1017/s1366728908003283

[ref39] Pérez-Sánchez M. A. Stadthagen-Gonzalez H. Guasch M. Hinojosa J. A. Fraga I. Marín J.. (2021). EmoPro - emotional prototypicality for 1286 Spanish words: relationships with affective and psycholinguistic variables. Behav. Res. Ther. 53, 1857–1875. doi: 10.3758/s13428-020-01519-9, PMID: 33629205

[ref40] Pessoa L. Kastner S. Ungerleider L. G. (2002). Attentional control of the processing of neutral and emotional stimuli. Cogn. Brain Res. 15, 31–45. doi: 10.1016/s0926-6410(02)00214-8 12433381

[ref41] Pintzinger N. M. Pfabigan D. M. Pfau L. Kryspin-Exner I. Lamm C. (2017). Temperament differentially influences early information processing in men and women: preliminary electrophysiological evidence of attentional biases in healthy individuals. Biol. Psychol. 122, 69–79. doi: 10.1016/j.biopsycho.2016.07.007, PMID: 27396749

[ref42] Pool E. Brosch T. Delplanque S. Sander D. (2016). Attentional bias for positive emotional stimuli: a meta-analytic investigation. Psychol. Bull. 142, 79–106. doi: 10.1037/bul0000026, PMID: 26390266

[ref43] Poulsen C. Luu P. Davey C. Tucker D. M. (2005). Dynamics of task sets: evidence from dense-array event-related potentials. Cogn. Brain Res. 24, 133–154. doi: 10.1016/j.cogbrainres.2005.01.008, PMID: 15922166

[ref44] Pourtois G. Schettino A. Vuilleumier P. (2013). Brain mechanisms for emotional influences on perception and attention: what is magic and what is not. Biol. Psychol. 92, 492–512. doi: 10.1016/j.biopsycho.2012.02.007, PMID: 22373657

[ref45] Pratto F. John O. P. (1991). Automatic vigilance: the attention-grabbing power of negative social information. J. Pers. Soc. Psychol. 61, 380–391. doi: 10.1037/0022-3514.61.3.380, PMID: 1941510

[ref46] Price R. B. Kuckertz J. M. Siegle G. J. Ladouceur C. D. Silk J. S. Ryan N. D.. (2015). Empirical recommendations for improving the stability of the dot-probe task in clinical research. Psychol. Assess. 27, 365–376. doi: 10.1037/pas0000036, PMID: 25419646 PMC4442069

[ref47] Schindler S. Kissler J. (2016). Selective visual attention to emotional words: early parallel frontal and visual activations followed by interactive effects in visual cortex. Hum. Brain Mapp. 37, 3575–3587. doi: 10.1002/hbm.23261, PMID: 27218232 PMC6867492

[ref48] Shushakova A. Wiesner C. D. Ohrmann P. Pedersen A. (2018). Electrophysiological evidence of an attentional bias towards appetitive and aversive words in adults with attention-deficit/hyperactivity disorder. Clin. Neurophysiol. 129, 1937–1946. doi: 10.1016/j.clinph.2018.06.019, PMID: 30007893

[ref49] Smith S. D. Bulman-Fleming M. B. (2005). An examination of the right-hemisphere hypothesis of the lateralization of emotion. Brain Cogn. 57, 210–213. doi: 10.1016/j.bandc.2004.08.046, PMID: 15708218

[ref50] Sutton T. M. Altarriba J. (2011). The automatic activation and perception of emotion in word processing: evidence from a modified dot probe paradigm. J. Cogn. Psychol. 23, 736–747. doi: 10.1080/20445911.2011.554392

[ref51] van Heck C. H. Oosterman J. M. de Kleijn K. M. A. Jongsma M. L. A. van Rijn C. M. (2017). Evidence for a priori existence of attentional bias subgroups in emotional processing of aversive stimuli. Front. Behav. Neurosci. 11:87. doi: 10.3389/fnbeh.2017.00087, PMID: 28553210 PMC5427543

[ref52] van Rooijen R. Ploeger A. Kret M. E. (2017). The dot-probe task to measure emotional attention: a suitable measure in comparative studies? Psychon. Bull. Rev. 24, 1686–1717. doi: 10.3758/s13423-016-1224-1, PMID: 28092078

[ref53] Vinson D. Ponari M. Vigliocco G. (2014). How does emotional content affect lexical processing? Cognit. Emot. 28, 737–746. doi: 10.1080/02699931.2013.851068, PMID: 24215294 PMC3979450

[ref54] Vuilleumier P. (2005). How brains beware: neural mechanisms of emotional attention. Trends Cogn. Sci. 9, 585–594. doi: 10.1016/j.tics.2005.10.011, PMID: 16289871

[ref55] Wang X. Shangguan C. Lu J. (2019). Time course of emotion effects during emotion-label and emotion-laden word processing. Neurosci. Lett. 699, 1–7. doi: 10.1016/j.neulet.2019.01.028, PMID: 30677433

[ref56] Wu C. Zhang J. (2019). Conflict processing is modulated by positive emotion word type in second language: an ERP study. J. Psycholinguist. Res. 48, 1203–1216. doi: 10.1007/s10936-019-09653-y, PMID: 31317377

[ref57] Wu C. Zhang J. Yuan Z. (2020a). An ERP investigation on the second language and emotion perception: the role of emotion word type. Int. J. Biling. Educ. Biling. 25, 539–551. doi: 10.1080/13670050.2019.1703895

[ref58] Wu C. Zhang J. Yuan Z. (2020b). Affective picture processing is modulated by emotion word type in masked priming paradigm: an event-related potential study. J. Cogn. Psychol. 32, 287–297. doi: 10.1080/20445911.2020.1745816

[ref59] Wu C. Zhang J. Yuan Z. (2021). Exploring affective priming effect of emotion-label words and emotion-laden words: an event-related potential study. Brain Sci. 11:553. doi: 10.3390/brainsci11050553, PMID: 33925670 PMC8145978

[ref60] Yiend J. (2010). The effects of emotion on attention: a review of attentional processing of emotional information. Cognit. Emot. 24, 3–47. doi: 10.1080/02699930903205698

[ref61] Yuan J. Tian Y. Huang X. Fan H. Wei X. (2019). Emotional bias varies with stimulus type, arousal and task setting: meta-analytic evidences. Neurosci. Biobehav. Rev. 107, 461–472. doi: 10.1016/j.neubiorev.2019.09.035, PMID: 31557549

[ref62] Zhang X. Dong Y. Zhou R. (2018). Examination stress results in attentional bias and altered neural reactivity in test-anxious individuals. Neural Plast. 2018:3281040. doi: 10.1155/2018/3281040, PMID: 29755511 PMC5884033

[ref63] Zhang D. He W. Wang T. Luo W. Zhu X. Gu R.. (2014). Three stages of emotional word processing: an ERP study with rapid serial visual presentation. Soc. Cogn. Affect. Neurosci. 9, 1897–1903. doi: 10.1093/scan/nst188, PMID: 24526185 PMC4249467

[ref64] Zhang J. Teo T. Wu C. (2019a). Emotion words modulate early conflict processing in a flanker task: differentiating emotion-label words and emotion-laden words in second language. Lang. Speech 62, 641–651. doi: 10.1177/0023830918807509, PMID: 30354948

[ref65] Zhang J. Wu C. Meng Y. Yuan Z. (2017). Different neural correlates of emotion-label words and emotion-laden words: an ERP study. Front. Hum. Neurosci. 11:455. doi: 10.3389/fnhum.2017.00455, PMID: 28983242 PMC5613167

[ref66] Zhang J. Wu C. Yuan Z. Meng Y. (2019b). Differentiating emotion-label words and emotion-laden words in emotion conflict: An ERP study. Exp. Brain Res. 237, 2423–2430. doi: 10.1007/s00221-019-05600-4, PMID: 31302735

[ref67] Zhang J. Wu C. Yuan Z. Meng Y. (2020). Different early and late processing of emotion-label words and emotion-laden words in second language: an ERP study. Sec. Lang. Res. 36, 399–412. doi: 10.1177/0267658318804850

[ref68] Zhao J. Li S. Lin S. E. Cao X. H. He S. Weng X. C. (2012). Selectivity of N170 in the left hemisphere as an electrophysiological marker for expertise in reading Chinese. Neurosci. Bull. 28, 577–584. doi: 10.1007/s12264-012-1274-y, PMID: 23054635 PMC5561926

[ref69] Zhong M. Zhu X. Yi J. Yao S. Atchley R. A. (2011). Do the early attentional components of ERPs reflect attentional bias in depression? It depends on the stimulus presentation time. Clin. Neurophysiol. 122, 1371–1381. doi: 10.1016/j.clinph.2010.09.016, PMID: 20961804

