# Supplementary material for: Evidence for dynamic attentional bias toward positive emotion-laden words: A behavioral and electrophysiological study
Source: Front Psychol. 2022 Aug 16;13:966774. doi: 10.3389/fpsyg.2022.966774 (PMC9426460; doi:10.3389/fpsyg.2022.966774)
Supplement: Supplementary file 1 [file Data_Sheet_1.docx]

**SUPPLEMENTARY MATERIAL**

The results of statistical analysis of the properties of materials are showed in the **Table 1c-e**.

**Table 1c**. The results of independent *t* test of words in positive and the first neutral word pair

|  | **P E-label Word Pair** | | |  | **P E-laden Word Pair** | | |  | **Neutral Word Pair1** | | |
| --- | --- | --- | --- | --- | --- | --- | --- | --- | --- | --- | --- |
|  | *df* | *t* | *p* |  | *df* | *t* | *p* |  | *df* | *t* | *p* |
| Arousal | 64 | 10.767 | <.001*** |  | 64 | 10.267 | <.001*** |  | 64 | 0.439 | .662 |
| Pleasantness | 64 | 14.128 | <.001*** |  | 64 | 13.007 | <.001*** |  | 64 | 1.331 | .188 |
| Abstractness | 64 | 1.245 | .218 |  | 64 | 0.253 | .801 |  | 64 | 0.891 | .377 |
| Stokes | 64 | 0.767 | .446 |  | 64 | 0.357 | .722 |  | 64 | 0.035 | .972 |
| Frequency | 64 | −1.401 | .166 |  | 64 | −0.809 | .422 |  | 64 | −1.428 | .158 |

Note: P = Positive, E-label = Emotion-label, E-laden = Emotion-laden

**Table 1d**. The results of independent *t* test of words in negative and the second neutral word pair

|  | **N E-label Word Pair** | | |  | **N E-laden Word Pair** | | |  | **Neutral Word Pair2** | | |
| --- | --- | --- | --- | --- | --- | --- | --- | --- | --- | --- | --- |
|  | *df* | *t* | *p* |  | *df* | *t* | *p* |  | *df* | *t* | *p* |
| Arousal | 64 | 13.895 | <.001*** |  | 64 | 13.859 | <.001*** |  | 64 | −1.003 | .319 |
| Pleasantness | 64 | −18.187 | <.001*** |  | 64 | −19.549 | <.001*** |  | 64 | −1.589 | .117 |
| Abstractness | 64 | 1.532 | .131 |  | 64 | 1.461 | .149 |  | 64 | −0.480 | .633 |
| Stokes | 64 | 1.565 | .122 |  | 64 | 0.062 | .951 |  | 64 | 0.609 | .545 |
| Frequency | 64 | −1.774 | .081 |  | 64 | 0.144 | .886 |  | 64 | −0.814 | .419 |

Note: N = Negative, E-label = Emotion-label, E-laden = Emotion-laden

**Table 1e**. The results of one-way ANOVA of the four types of emotional words and neutral words

|  | ***F* (4, 391)** | ***p*** |
| --- | --- | --- |
| Arousal | 164.160 | <.001*** |
| Pleasantness | 534.268 | <.001*** |
| Abstractness | 1.964 | .099 |
| Stokes | 0.590 | .670 |
| Frequency | 1.384 | .239 |

The emotional words used in the experiment.

| positive emotion-label words | positive emotion-laden words | negative emotion-label words | negative emotion-laden words |
| --- | --- | --- | --- |
| 欢畅 | 卓越 | 悲叹 | 潦倒 |
| 欣慰 | 壮观 | 悲苦 | 荒诞 |
| 畅快 | 提升 | 恐吓 | 低劣 |
| 狂喜 | 辉煌 | 轻视 | 阴暗 |
| 欢欣 | 甘甜 | 愤恨 | 出卖 |
| 真挚 | 壮丽 | 悲怆 | 荒唐 |
| 赞许 | 融洽 | 伤感 | 淫秽 |
| 舒畅 | 雄伟 | 失意 | 缺乏 |
| 赞扬 | 甜美 | 惶恐 | 险恶 |
| 振奋 | 亲近 | 悲惨 | 卑劣 |
| 喜乐 | 改善 | 憎恨 | 贪婪 |
| 珍爱 | 优美 | 威慑 | 艰苦 |
| 亢奋 | 兴旺 | 内疚 | 嚣张 |
| 欢愉 | 生动 | 忧郁 | 奸诈 |
| 欢快 | 富贵 | 疑虑 | 恶毒 |
| 欣喜 | 豪放 | 悲哀 | 虚伪 |
| 快活 | 亲热 | 消极 | 虚假 |
| 欣赏 | 光荣 | 消沉 | 凄凉 |
| 敬重 | 精彩 | 悲愤 | 挑衅 |
| 依恋 | 活泼 | 仇恨 | 懒惰 |
| 轻松 | 温暖 | 嫉妒 | 衰老 |
| 着迷 | 亲密 | 忧愁 | 可耻 |
| 逗乐 | 优秀 | 厌倦 | 恶劣 |
| 激励 | 浪漫 | 惆怅 | 贫穷 |
| 热情 | 信赖 | 悲观 | 质疑 |
| 欢乐 | 勤奋 | 懊恼 | 丑陋 |
| 欢喜 | 幽默 | 抱怨 | 逃避 |
| 崇拜 | 孝顺 | 责怪 | 错误 |
| 愉悦 | 支持 | 傲慢 | 拒绝 |
| 爱惜 | 信任 | 压抑 | 苛刻 |
| 尊重 | 美丽 | 讨厌 | 排斥 |
| 积极 | 慰问 | 悲伤 | 放弃 |
| 享受 | 圣洁 | 郁闷 | 责备 |
